# Inhibition of KIF23 Alleviates IPAH by Targeting Pyroptosis and Proliferation of PASMCs

**DOI:** 10.3390/ijms23084436

**Published:** 2022-04-18

**Authors:** Zeang Wu, Guangyuan Zhou, Haixia Wang, Ping Yao

**Affiliations:** 1 School of Public Health, Tongji Medical College, Huazhong University of Science and Technology, Wuhan 430030, China; wuzeang88@163.com; 2 School of Basic Medicine, Tongji Medical College, Huazhong University of Science and Technology, Wuhan 430030, China; 18770227768@163.com; 3 School of Medicine, Shihezi University, Shihezi 832002, China; wanghaixiawza@163.com

**Keywords:** KIF23, PASMCs, IPAH, proliferation, pyroptosis

## Abstract

Idiopathic pulmonary arterial hypertension (IPAH) is a progressive vascular disease with high mortality and heritability. Pyroptosis is a novel form of programmed cell death, and it is closely associated with IPAH. However, the roles of pyroptosis-related genes (PRGs) in IPAH are still largely unknown. In this study, we identified KIF23 as the most relevant gene for IPAH and pyroptosis, and its expression was significantly increased in pulmonary arterial smooth muscle cells (PASMCs) of IPAH. Besides, the pyroptosis level of PASMCs was also considerably upregulated in IPAH. Knockdown of KIF23 in PASMCs could significantly suppress the PASMCs’ pyroptosis and proliferation and then alleviate the increase in pulmonary arterial pressure, right ventricular hypertrophy, and pulmonary vascular resistance in IPAH. KIF23 regulated the expression of Caspase3, NLRP3, and HMGB1, and they were all involved in the PI3K/AKT and MAPK pathways, indicating that PI3K/AKT and MAPK pathways might participate in regulating PASMCs pyroptosis by KIF23. In conclusion, our study suggests that KIF23 may be a new therapeutic target for IPAH, which can alleviate the symptoms of IPAH by inhibiting the pyroptosis and proliferation of PASMCs.

## 1. Introduction

Idiopathic pulmonary arterial hypertension was a devastating pulmonary vascular disease mainly caused by arteriosclerosis, stenosis, embolism, and vascular remodeling, leading to right heart failure and death. The median survival and 5-year survival rates of IPAH patients were 2.8 years and 34%, respectively [1]. The current therapeutic drugs mainly include calcium channel blockers [2], prostacyclins [3], statins [4], nitric oxide [5], endothelin receptor antagonists [6], and 5-phosphodiesterase inhibitors [7]. Lung transplantation was currently the only effective treatment for IPAH, and many IPAH patients could not receive lung transplantation due to insufficient donors. Therefore, it is essential to deeply study the pathogenesis of IPAH and develop new therapeutic targets.

Emerging evidence suggested the contribution of PASMCs to IPAH pathogenesis. PASMCs of IPAH patients showed cancer-like characteristics, including excessive proliferation and apoptosis resistance, which were crucial mechanisms of IPAH development [8,9,10]. Meanwhile, hypertrophy, obstructive hyperplasia, and phenotypic switching of PASMCs in patients with IPAH can also lead to increased pulmonary vascular resistance and pulmonary artery pressure [11,12]. Multiple studies confirmed that targeting genes responsible for abnormal features in PASMCs contributed to developing new treatments for IPAH [13,14,15]. Besides, as a physiological regulator of cell proliferation, different forms of programmed cell death (PCD) in PASMCs were also reported to be associated with IPAH, including apoptosis [16], autophagy [17], necrosis [18], and pyroptosis [19]. Therefore, exploring new target genes involved in PCD can provide more options for diagnosing and treating IPAH disease.

As a new inflammatory form of cell death, pyroptosis is triggered by caspase-1/4/5/11 mediated by some inflammasome. Then it could result in the cleavage of Gasdermin D (GSDMD) and cause cell perforation and death. It releases a large amount of pro-inflammatory factors such as IL-18 and IL-1β. Studies have shown that pyroptosis can inhibit the proliferation of cancer cells and promote the inflammatory cell death of cancer cells [20]. Pyroptosis plays an essential role in vascular inflammation and cardiovascular disease [21]. In hypoxia and monocrotaline (MCT) induced animal models of pulmonary hypertension, the media of the pulmonary artery [22]. The pyroptosis-related genes were also remarkably increased in human PASMCs under hypoxic conditions, including HMGB1, IL-18, and caspase-1 [23]. Thence, we hypothesized that PASMCs in IPAH may also undergo pyroptosis. It is of great importance to reveal the role of pyroptosis in IPAH.

This study calculated the pyroptosis enrichment score for each sample using bioinformatics methods and divided all samples into two subgroups. Then, we used the weighted gene co-expression network (WGCNA) to screen the hub gene (KIF23) most related to IAPH and pyroptosis. Next, we analyzed the biological functions and signaling pathways involved in KIF23. Finally, we examined the expression levels of KIF23 and PRGs in PASMCs from control and IPAH animals and the altered expression levels of KIF23 and pyroptosis-related genes after knockdown of KIF23 expression. Our research results provide new ideas for understanding the impact of KIF23 on PASMCs pyroptosis and IPAH.

## 2. Results

### 2.1. Screen for DEGs between Control and IPAH Samples

**Figure 1:**
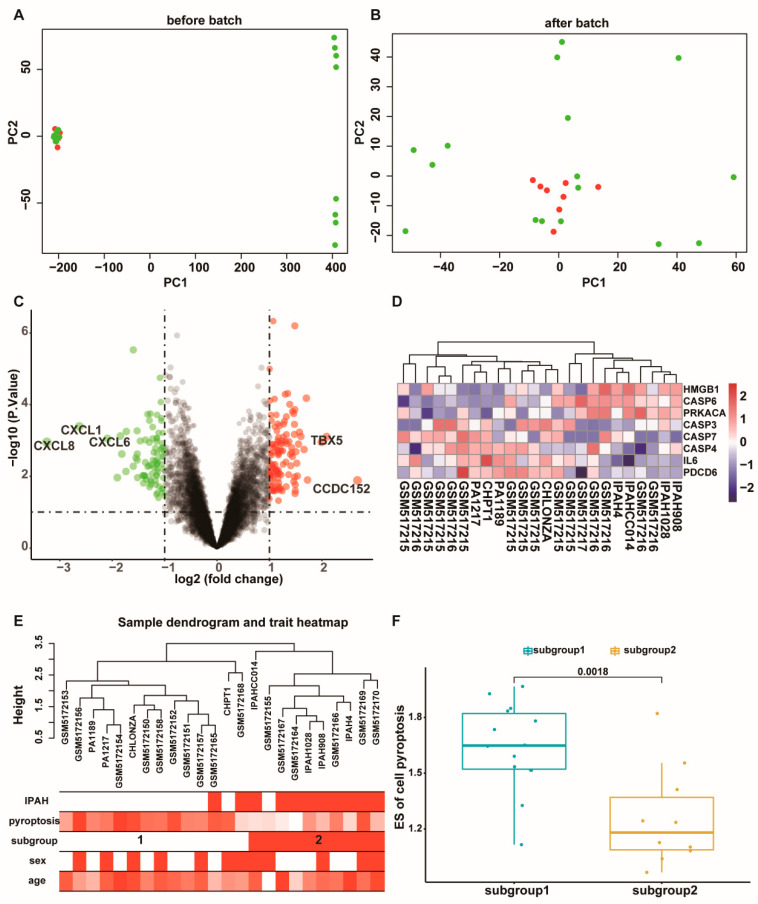
Data processing and sample clustering. (**A**,**B**) Principal Components Analysis of all samples before and after removing batch effect. (**C**) Volcano plot of all the gene expression altered in IPAH compared to the control samples. (**D**) The Heatmap plot showed the expression of 8 PRGs in each sample. (**E**) Sample clustering was performed based on the expression of 8 PRGs between IPAH and control samples. Color intensity was proportional to IPAH status, the ES of pyroptosis, subgroup, sample age, and gender. (**F**) The ES of pyroptosis between subgroups.

A total of 12,970 common genes were filtered from GSE144274 and GSE168905 databases after normalization. We found a batch effect between the two databases and then removed the batch effect (Figure 1A,B and Figure S1A). According to the filter criteria we set, 198 significant DEGs, including 116 upregulated and 82 downregulated DEGs, were screened out between IPAH and control samples. We showed the expression patterns of all genes in the volcano plot and marked the gene symbol of DEGs with |log2fold change(FC)| > 3 in the volcano plot (Figure 1C). We examined the expression of 56 PRGs and found 8 PRGs significantly differentially expressed between IPAH and control samples. A heat map of these eight PRGs was conducted and shown in Figure 1D. According to these eight PRGs, all samples were divided into two subgroups (Figure 1E). Subgroup1 was mainly composed of control samples, and that subgroup2 was mostly of IPAH samples. We calculated the cell pyroptosis ES of each sample with the ssGSEA method and compared the difference in the cell pyroptosis ES between subgroups. The ES of cell pyroptosis was significantly increased in subgroup1 compared with subgroup2 (Figure 1F).

### 2.2. Construct the Co-Expression Network and Identify IPAH Related Modules

**Figure 2:**
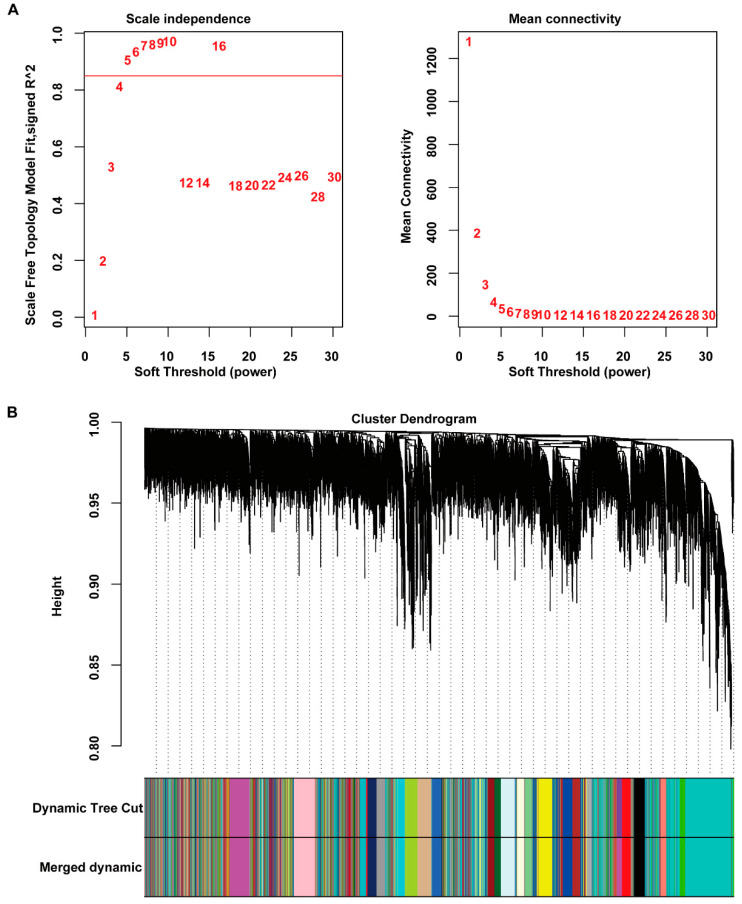
Construction of the weighted gene co-expression network. (**A**) Use soft threshold power analysis to obtain the scale-free fit index of the network topology. (**B**) A hierarchical cluster analysis was performed to detect co-expression clusters with corresponding color assignments. Each color represents a module in the gene co-expression network constructed by WGCNA.

We selected all genes to construct the co-expression network to reduce the adverse effect of artificially setting screening criteria on the DEGs. All samples were performed a cluster analysis to detect outliers, and we found no outliers. The soft threshold power of β was five (scale independence = 0.9) based on the scale-free topology criterion (Figure 2A). We checked the scale-free topology when soft threshold power β = 5 and found that the selected β value could establish a scale-free network of genes (scale-free R2 = 0.85, Figure S1B). According to our screening criteria, we identified 24 modules in the network (Figure 2B) and calculated the gene number of each module (Table S3). We placed genes that could not be included in any module in the gray module and deleted them in the following analysis.

**Figure 3:**
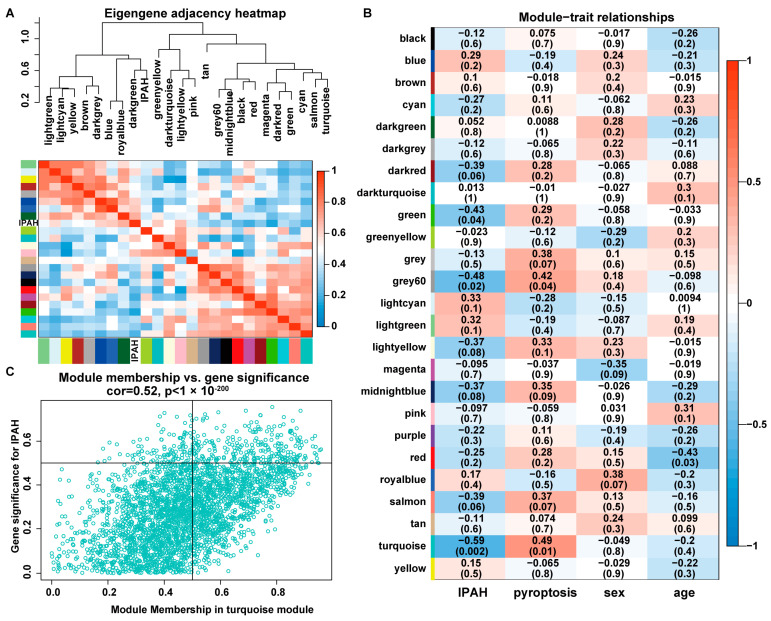
Correlation of modules with IPAH. (**A**) Module feature gene dendrogram and feature gene network heat map analysis of the correlation of the modules generated in WGCNA. (**B**) Correlation analysis of the modules and clinical traits. (**C**) Correlation between MM and GS in the turquoise module.

To analyze the interaction association of 24 modules, we calculated the eigengenes of 24 modules and clustered them according to their correlations to quantify their co-expression similarity. The eigengene adjacency heatmap separated these 24 modules into two meta-modules (Figure 3A). The first meta-modules contained eight modules that were mainly positively related to IPAH. The second meta-modules included 15 modules that were mainly negatively related to IPAH. Among these 23 modules, the turquoise module (3125 genes) was the most negatively (R = −0.59, *p* = 0.002) module associated with IPAH and positively (R = 0.49, *p* = 0.01) module related to cell pyroptosis (Figure 3B). To search for the most important genes related to IPAH in the module, we performed the intramodular analysis for gene significance (GS) and module membership (MM). 309 hub genes were screened out based on the threshold abs(cor. MM) > 0.5, abs(cor. GS) > 0.5, and *p* value < 0.05 (Figure 3C). These hub genes were chosen as the target of subsequent analysis.

### 2.3. Identify Top 10 Hub Genes in the Turquoise Module

**Figure 4:**
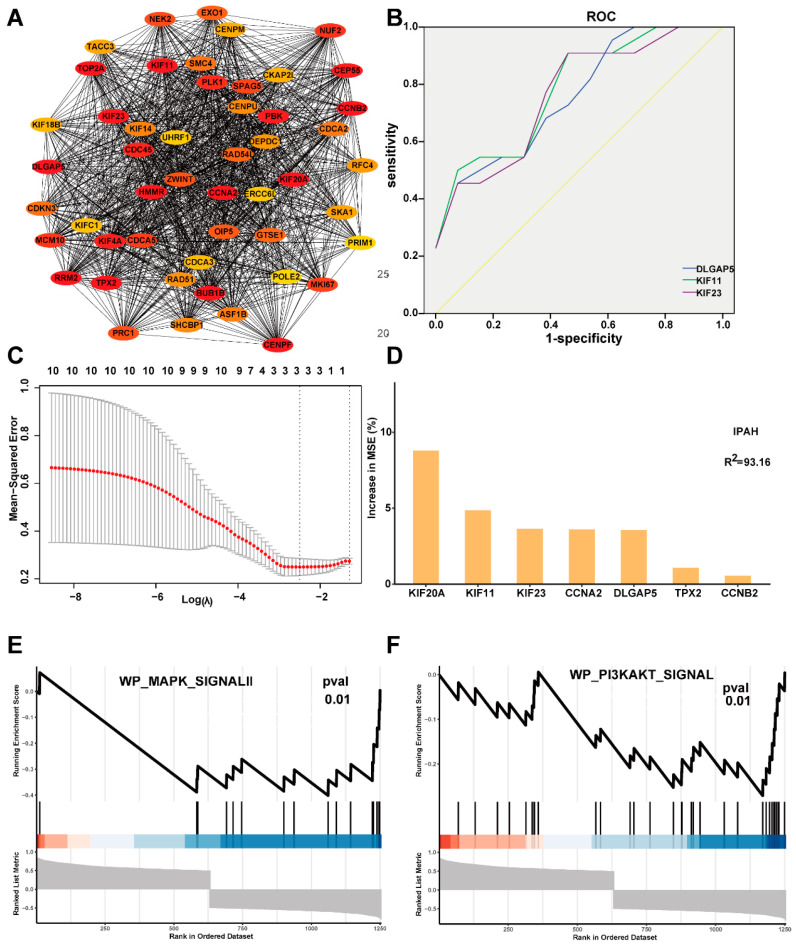
Identify the hub gene most related to IPAH and pyroptosis in the turquoise module. (**A**) The PPI network of top 50 hub gens. (**B**) ROC curves of top 10 hub genes. (**C**) LASSO regression of the top 10 IPAH-related genes. (**D**) SVM-RFE regression of the top 10 IPAH-related genes. (**E**) The enrichment plot of MAPK pathway. (**F**) The enrichment plot of PI3K/AKT pathway.

The PPI network of the turquoise module contained 241 nodes and 2468 edges. We screened out the top 10 hub genes with the MCC method of cytoHubba Plug-in (Table 1), and the PPI network of the top 10 hub genes with neighbors was shown in Figure 4A.

MOCDE Plug-in screened out nine clusters in the turquoise module. In the turquoise module, cluster 1 (58 genes) was mainly associated with DNA replication, cell cycle, and mismatch repair. Cluster 2 (5 genes) was mainly associated with the chemokine signaling pathway and cytokine–cytokine receptor interaction.

### 2.4. Evaluate the Diagnostic and Prognostic Value of the Top 10 Hub Genes

We performed ROC analysis to evaluate the diagnostic ability of the top 10 hub genes and found that three hub genes have good diagnostic value. The Area Under the Curve (AUC) of DLGAP5, KIF11, and KIF23 were 0.766 (95% CI:0.574–0.957), 0.790 (95% CI:0.606–0.974), and 0.773 (95% CI:0.571–0.964), respectively (Figure 4B). The optimum λ value was three in the LASSO Cox regression analysis, and we included three genes to construct a prognostic gene model. The risk score = 0.01*DLGAP5 + 0.037*KIF23 +0.115*KIF20A. The top three feature genes returned by randomForest Cox regression analysis were KIF20A, KIF11, and KIF23 (Figure 4C,D). KIF23 was the only common gene in all analysis results, which was differentially expressed between IPAH and control samples (*p* < 0.05), so we chose KIF23 as the follow-up analysis target.

### 2.5. KIF23 Was Involved in the MAPK and PI3K/AKT Signaling Pathways

After calculating the relationship between KIF23 and all other genes, 1300 genes that significantly correlated to KIF23 were filtered. To explore the potential biological functions of KIF23, we performed the GSEA analysis on the 1300 genes. A total of 106 pathways positively correlated to KIF23, while 22 negatively correlated to KIF23. Among them, the MAPK and PI3K/AKT pathways, which have been reported to be involved in the regulation of pyroptosis, were significantly negatively correlated with KIF23 (Figure 4E,F).

### 2.6. KIF23 and Pyroptosis Levels Were Increased in PASMCs of IPAH

**Figure 5:**
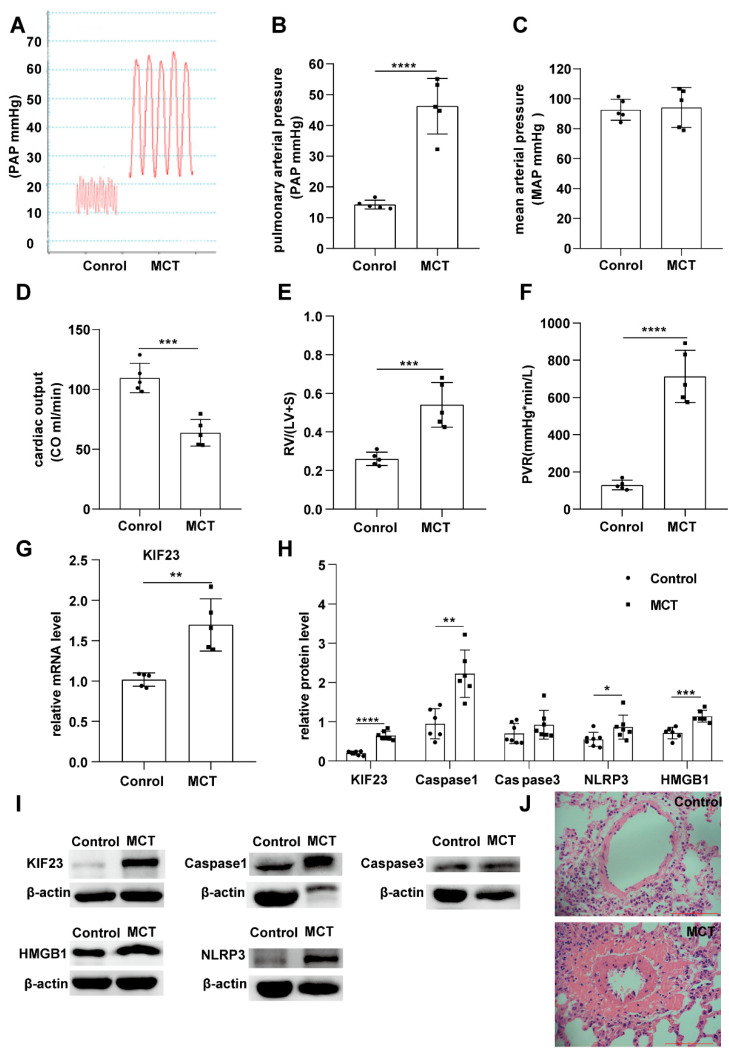
Identify the expression of KIF23 in the MCT-induced IPAH animal module. (**A**–**F**) Present the pulmonary arterial pressure, mean arterial pressure, cardiac out, RV/(LV + S), CO, and peripheral vascular resistance in Control and MCT groups. (**G**) The relative mRNA level of KIF23 between Control and MCT groups. (**H**,**I**) The protein level of KIF23, Caspase1, Caspase3, NLRP3, and HMGB1 between the Control and MCT groups. *n* = 5 per group. * *p* < 0.05, ** *p* < 0.01, *** *p* < 0.001, **** *p* < 0.0001 versus Control. Data are mean ± SEM. (**J**) HE staining a picture of the pulmonary artery.

Compared to control rats, pulmonary arterial pressure, right ventricular hypertrophy, and pulmonary vascular resistance were significantly increased, and the cardiac output was reduced considerably in IPAH rats (Figure 5A–F). HE staining showed that the pulmonary arterioles of IPAH rats were narrowed, smooth muscle cells in the vascular media increased, and the vessel wall thickened. All parameters indicated that the IPAH animal model had been successfully established.

We tested the expression of KIF23 in the IPAH animal module and found that KIF23 was significantly upregulated in IPAH rats. Meanwhile, The protein levels of KIF23 and PRGs (caspase1, caspase3, NLRP3, and HMGB1) were also increased dramatically in IPAH (Figure 5G–J), these results indicating that KIF23 was involved in PASMCs pyroptosis and IPAH.

### 2.7. Knockdown KIF23 Alleviates IPAH by Targeting Pyroptosis and Proliferation in PASMCs

**Figure 6:**
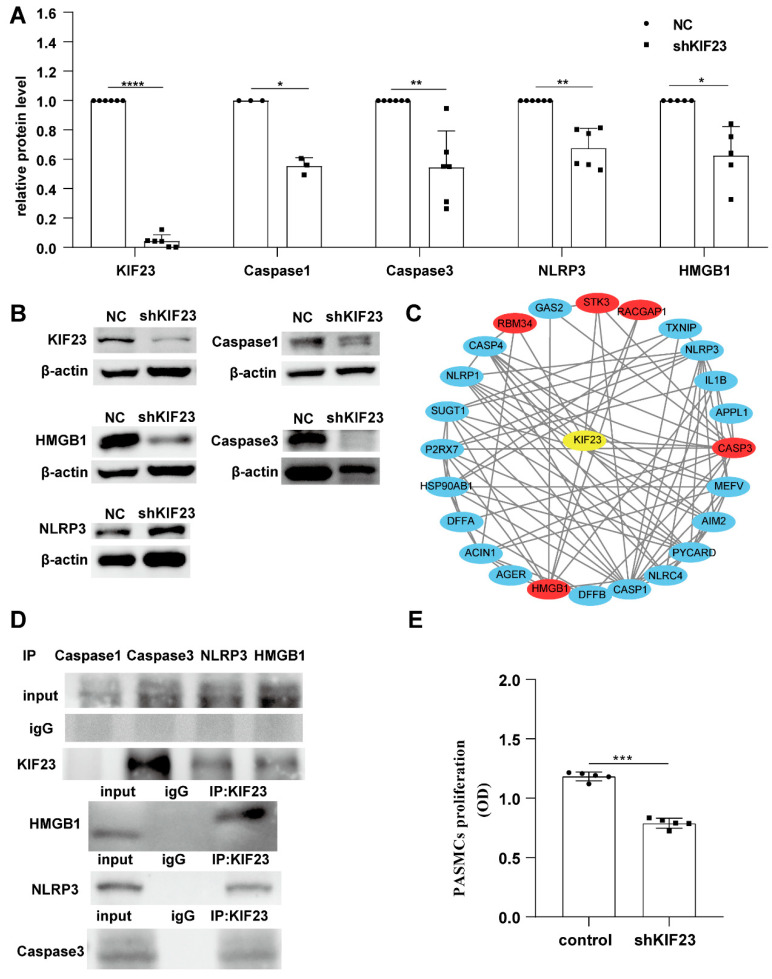
The effect of KIF23 knockdown on PASMCs pyroptosis. (**A**,**B**) The protein level of KIF23, Caspase1, Caspase3, NLRP3, and HMGB1 between NC and shKIF23 groups. *n* = 5 per group. * *p* < 0.05, ** *p* < 0.01, *** *p* < 0.001, **** *p* < 0.0001 versus NC. Data are mean ± SEM. (**B**) The predicted protein interactions of KIF23, Caspase3, NLRP3, and HMGB1. (**C**) PRGs that may interact with KIF23. (**D**) Co-IP results showed that KIF23 interact with Caspase3, NLRP3, and HMGB1. (**E**) The effect of KIF23 knockdown on PASMCs proliferation.

To explore the role of KIF23 in PASMCs pyroptosis, we knocked down the expression of KIF23 in PASMCs with shRNA and found the expression of KIF23 was drastically reduced. Suppression of KIF23 resulted in a significant reduction in caspase1, caspase3, NLRP3, and HMGB1, indicating that KIF23 was negatively regulated the pyroptosis of PASMCs (Figure 6A,B).

**Figure 7:**
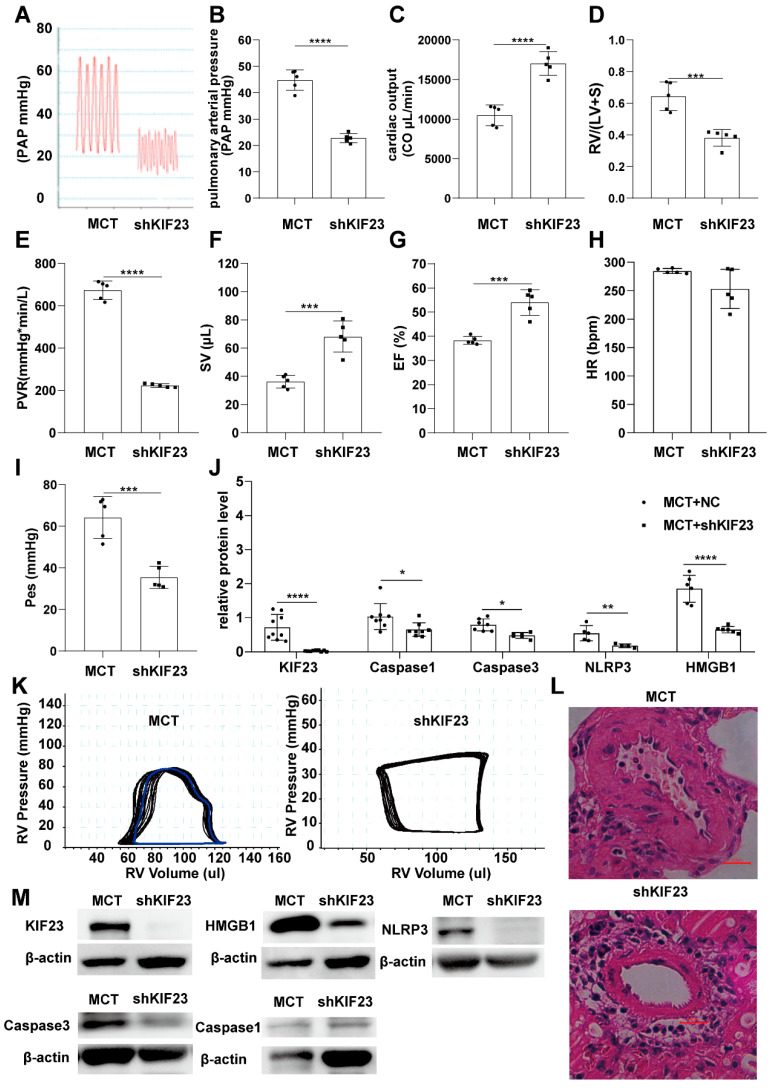
KIF23 knockdown alleviates the process of IPAH. (**A**–**I**) Present the pulmonary arterial pressure, cardiac out, RV/(LV + S), CO, peripheral vascular resistance, stroke volume, ejection fraction, heart rate, and end-systolic pressure in MCT and shKIF23 groups. (**J**) The protein level of KIF23, Caspase1, Caspase3, NLRP3, and HMGB1 between MCT and shKIF23 groups. (**K**) The PV Loop analysis of right ventricle between MCT and shKIF23 groups. *n* = 5 per group. * *p* < 0.05, ** *p* < 0.01, *** *p* < 0.001, **** *p* < 0.0001 versus MCT. Data are mean ± SEM. (**L**) HE staining a picture of the pulmonary artery. (**M**) The protein level of KIF23, Caspase1, Caspase3, NLRP3, and HMGB1 between MCT and shKIF23 groups.

We next predicted the possibility that these four PRGs interact with KIF23 and found that caspase3 and HMGB1 might interact with KIF23. Surprisingly, Co-IP results demonstrated that Caspase3, NLRP3, and HMGB1 interacted with KIF23 (Figure 6C,D). Some studies have found that KIF23 has a role in regulating cell proliferation in various cancer, so we speculate that KIF23 may regulate the proliferation of PASMCs in PAH. CCK-8 assay showed that KIF23 knockdown significantly suppressed PASMCs proliferation (Figure 6E). Since KIF23 was significantly upregulated in IPAH, we tried to test whether KIF23 knockdown would inhibit the progression of IPAH. Compared with MCT-induced IPAH rats, KIF23 knockdown significantly reduced the pulmonary arterial pressure, right ventricular hypertrophy, pulmonary vascular resistance, end-systolic pressure, and pulmonary vascular remodeling. At the same time, it also maintained the cardiac output, right ventricular volume, and ejection fraction. HE staining results indicated that KIF23 knockdown prevented the vascular remodeling in IPAH (Figure 7A–L). Overall, these results showed that KIF23 suppression alleviated IPAH progression. To explore whether KIF23 was involved in regulating PASMCs pyroptosis at the animal level, we next tested the protein level of caspase1, caspase3, NLRP3, and HMGB1 in IPAH after suppressing KIF23. Interestingly, the pyroptosis level of PASMCs was sharply reduced in KIF23 knockdown rats compared to the MCT group (Figure 7M), demonstrating that a high level of KIF23 was essential to the pyroptosis of PASMCs.

## 3. Discussion

Cell proliferation and PCD play an essential role in maintaining biological development and homeostasis. Excessive cell proliferation and abnormal cell death could lead to various diseases. For example, excessive proliferation [24] and anti-apoptosis [25] of PASMCs were very typical pathological characteristics of PAH. PCD is mainly composed of apoptosis, necrosis, and pyroptosis. Pyroptosis was a form of lytic PCD caused by inflammasomes, different from apoptosis and necrosis. Classical and non-classical signaling pathways executed pyroptosis; both could cleave GSDMD protein and release IL1β, IL18, and other inflammatory factors. The N- and C-terminal fragments of GSDMD could induce cell membrane perforation and cell rupture [26]. Only one study has confirmed that pyroptosis occurred in PASMCs of hypoxia-induced PH and MCT-induced PAH mice [23]. However, the mechanism of pyroptosis in IPAH is still largely unknown. The exploration of potential target genes involved in pyroptosis is of great significance to the treatment of IPAH.

In this paper, we confirmed that KIF23 was significantly correlated to the regulation of pyroptosis and IPAH. KIF23 is a nuclear protein that regulates cytokinesis and microtubule-binding and plays a crucial role in mitosis, intracellular vesicle transport, and membrane organelle positioning. Although there are no related reports on the direct study of the relationship between KIF23 and PAH, several studies also predicted KIF23 as the hub gene of IPAH [27,28]. As far as we know, the present study was the first to discover the role of KIF23 in pyroptosis and IPAH. KIF23 was significantly upregulated in PASMCs of IPAH rats, and KIF23 knockdown in PASMCs could alleviate the progress of IPAH, demonstrating that a high expression level of KIF23 was essential to the development of IPAH.

There are pieces of evidence that showed the vital role of PASMCs pyroptosis in hypoxia- and MCT-induced PH models [23]. Our study also found the expression of several PRGs (Caspase1, Caspase3, NLRP3, and HMGB1) were significantly increased in PASMCs of IPAH, once again confirming that PASMCs undergo pyroptosis in IPAH. WGCNA analysis suggested that KIF23 was closely related to pyroptosis, indicating that KIF23 might affect IPAH by regulating PASMCs pyroptosis. In PASMCs, knockdown of KIF23 with shRNA resulted in significantly reduced expression of several PRGs. This negative regulation of pyroptosis by KIF23 was also observed in PASMCs from IPAH rats with knockdown of KIF23. To sum up, our findings extended a potentially important function of KIF23 in PASMCs pyroptosis and provided a new target for the prevention and treatment of IPAH.

To investigate which signaling pathway was involved in regulating PASMCs pyroptosis and IPAH by KIF23, we performed GSEA on KIF23-related genes and found that these genes were enriched in PI3K/AKT and MAPK pathways. Existing studies have shown that PI3K/AKT and MAPK signaling pathways were involved in pyroptosis and PAH. PI3K/AKT pathways were activated in the livers and renal tubular epithelial cells of ducks and subsequently triggered the process of cell pyroptosis [29,30]. The PI3K/AKT pathway was involved in regulating pyroptosis, and its role in PAH has also been confirmed. Dioscin [31] and Luteolin [32] could inhibit the PASMCs proliferation and migration by adjusting the PI3K/AKT pathway.

Similarly, inhibition of the MAPK pathway could suppress pyroptosis of several cell types [33,34,35]. Studies also showed that the MAPK pathway regulates the proliferation of PASMCs in PAH [36,37,38].

KIF23 regulated the expression of Caspase3, NLRP3, and HMGB1, and they also interacted with KIF23. Besides, they were all involved in the PI3K/AKT and MAPK signaling pathways, which were closely related to KIF23. HMGB1-triggered inflammation was associated with the MAPK pathway [39], and the HMGB1/RAGE/PI3K/Akt pathway was involved in regulating cell proliferation and autophagy in pancreatic cancer [40]. The activation of NLRP3 was regulated by PI3K or AKT in atherosclerosis [41], and MAPK/NLRP3 inflammasome/STAT3 signaling was involved in the neuroinflammatory responses [42]. Inhibition of MAPK/caspase-3 could block the pyroptosis induced by reactive oxygen species in mouse adipose tissue [43]. Meanwhile, caspase-3 was downstream of PI3K/ AKT [44], and ROS/PTEN/PI3K/Akt/Caspase3 pathway was responsible for regulating cell proliferation and apoptosis in prostate cancer [45]. To sum up, KIF23 might regulate PASMCs pyroptosis in IPAH through PI3K/AKT and MAPK pathways, which involved Caspase3, NLRP3, and HMGB1.

KIF23 has been proven to promote cell proliferation in many cancer cells [46,47,48]. Given the previous findings, we tried to examine whether KIF23 regulates PASMCS proliferation in IPAH, and the CCK-8 assay confirmed the inhibitory effect of KIF23 knockdown on the proliferation of PASMCs. Adequate studies have shown that the hyperproliferation and anti-apoptosis of PASMCs are the main factors of pulmonary artery remodeling in IPAH [49]. Among many signaling pathways involved in the proliferation of PASMCs, some studies found that inhibition of PI3K/AKT and MAPK pathways can significantly inhibit the proliferation of PASMCs [50,51]. So, we speculate that KIF23 might regulate PASMCs proliferation in IPAH through PI3K/AKT and MAPK pathways.

However, this study still has some limitations. To obtain enough samples for WGCNA analysis, we combined the samples of the two databases. Although the batch effect is eliminated as much as possible, the impact of different sequencing platforms is inevitable. In addition, some conclusions of this study still need further in vivo and in vitro experimental verification to confirm the specific role of KIF23 in IPAH and pyroptosis.

## 4. Materials and Methods

### 4.1. Bioinformatics Analysis to Find Hub Genes

#### 4.1.1. Data Preprocessing and DEGs Screening

We included 11 IPAH and 13 control samples from GSE144274 and GSE168905 (Table S1) in our study, and the detailed information on each sample is in Table 2. We first used the limma R package [52] to process the gene expression matrix of GSE144274 and GSE168905, then merged the processed data by gene symbol and removed the batch effect by sva R package. We screened for differentially expressed genes (DEGs) between IPAH samples and control samples with the limma R package, the cut-off criteria for screening DEGs were *p* Value < 0.05 and |log_2_^fold change(FC)^| > 1.

#### 4.1.2. Calculation of the Enrichment Score of Cell Pyroptosis

We obtained 56 PRGs from published studies (Table S2). We first used the limma R package to screen the differentially expressed PRGs between IPAH and control PASMCs. Then we clustered all samples by the Hierarchical Clustering method according to the shared genes of DEGs and PRGs. Next, we constituted a reference gene set of cell pyroptosis with these genes and calculated the enrichment score (ES) of cell pyroptosis by GSVA [53] R package. Finally, we figured out the difference in the ES of cell pyroptosis between different subgroups.

#### 4.1.3. Weighted Gene Co-Expression Network Construction and Hub Genes Detection

We used the WGCNA R package [54] to construct a weighted gene co-expression network and identify IPAH and pyroptosis-related modules. We first checked the data quality of all samples and used a scale-free topology criterion to calculate the soft threshold power of β, which was used to construct a scale-free network. Then, we selected modules with >100 RNAs at the minimum cut height of 0.2 by the dynamic tree cut method. Lastly, we investigated the correlations between these modules and IPAH. Genes in the most significant IPAH-related module were selected to calculate their relationship with modules and IPAH. Genes with both gene significance (GS) and module membership (MM) greater than 0.5 were defined as the hub genes.

#### 4.1.4. Protein–Protein Interaction (PPI) Network Construction

The hub genes were submitted to the Search Tool for the Retrieval of Interacting Genes/Proteins (STRING, https://string-db.org/ (accessed on 12 September 2021) to construct the PPI network and further screened out the top hub genes, which were highly connected nodes with a more critical role in the whole network. We imported the nodes with a combined score greater than 0.4 into Cytoscape 3.80 software to construct a PPI network to obtain more reliable results. Then we screened for hub genes and analyzed the clusters in the PPI network by CytoHubba and MCODE plug-in.

#### 4.1.5. Evaluate the Diagnostic and prognostic VALUE of the Top 10 Hub Genes

To evaluate the capability of hub genes to distinguish between IPAH and control samples, we used the ROCR package to calculate the prediction accuracy of the top 10 hub genes and the disease risk score of all samples. We calculated the area under the ROC curve (AUC), and genes with larger AUC values can distinguish IPAH and control samples well. Then, we performed LASSO and randomForest Cox regression analysis to evaluate the prognostic significance of the PRGs by glmnet and rfPermute R packages, respectively. We selected the hub genes confirmed by the two methods for subsequent analysis.

#### 4.1.6. Gene Set Enrichment Analysis (GSEA) of Hub Genes

To further explore the biological functions of hub genes, we analyzed their correlation with all other genes. Genes with a *p* value less than 0.05 and a correlation coefficient more significant than 0.5 were included in the GSEA analysis. We regarded the correlation coefficient between each gene and hub genes as its logFC and used the clusterProfiler R package to perform GSEA analysis. The previously annotated gene set c2.cp.v7.4.symbols.gmt was chosen as the reference gene set, and the *p* value < 0.05 was significant.

### 4.2. In Vivo and In Vitro Experiments to Study the Role of Hub Gene in Pyroptosis and PAH

#### 4.2.1. Animals and Treatments

Twenty-four male Sprague Dawley rats were from Liaoning Changshegn biotechnology Co., Ltd. and randomly divided into four groups: control, MCT, MCT + NC, MCT + shRNA. Rats were raised under standard temperature, humidity, and normal circadian cycle conditions, with free access to food and water. The MCT and control groups were injected intraperitoneally with MCT (Sigma) and normal saline at a dose of 60 mg/kg, respectively. Then, transfection of NC and shRNA plasmids into PASMCs using lentiviral vectors. Three weeks later, we successfully established the animal disease model of IPAH. The ethical committee of Shihezi University approved all animal experiments.

#### 4.2.2. Hemodynamic Analysis

We detected the hemodynamic index of rats with the PowerLab system and Millar catheter. Briefly, the catheter was inserted into the jugular vein, measured pulmonary arterial pressure (PPA), then inserted Millar catheter into the ventricle to measure cardiac output, ventricular pressure, ejection fraction, and ventricular volume. At last, we calculated the right ventricular hypertrophy index.

#### 4.2.3. Short Hairpin RNA (shRNA) Design and Transfection

The short hairpin RNA was synthesized by WZBIO (Wuhan, China). The sense sequences of shRNA against KIF23 was 5′-GCGGACAATCGGTTCAGTTTA-3′ and we chose the non-targeted control shRNA as negative control (NC). PASMCs were cultured in a 6 cm cell culture dish at 60–80% confluence and then added 10 μL shRNA into the DMEM. We incubated the mixture at 37 °C for 48 h and then harvested the cells to experiment as needed.

#### 4.2.4. Antibodies

Antibodies against kinesin family member 23 (KIF23, ab174304) were purchased from Abcam. Antibodies against caspase-1 (sc-s92736) were purchased from SANTA. Antibodies against high mobility group box 1 (HMGB1, 10829-1-AP), β-actin (66009-1-LG), and caspase-3 (19677-1-AP) were purchased from Proteintech. Antibodies against the NLR family pyrin domain containing 3 (NLRP3, NBP2-1244633) were purchased from Novus.

#### 4.2.5. Culture of PASMCs

We dissected a healthy, 4-week-old male SD rat and removed the heart and lungs after anesthetized. Then, we stripped the pulmonary artery vascular endothelium, and adventitia with a cotton swab and separated the PASMCs by trypsin type I collagen and trypsin. PASMCs were cultured in DMEM (20% FBS) at the condition of 5% CO_2_ at 37 °C.

#### 4.2.6. RT-qPCR

We isolated the PASMCs and extracted RNA for RT-qPCR to verify the selected hub genes. The RNA extracted by Trizol reagents was reverse transcribed into cDNA by HiScript^®^ Ⅱ Q RT SuperMix (Vazyme, China). Quantitative real-time polymerase chain reaction (qRT-PCR) was performed with ChamQ SYBR qPCR Master Mix (Vazyme, China) in a Step One Plus real-time PCR system (Applied Biosystems QuantStudio Q7, USA). We used the 2−ΔΔCt method to quantify the expression of selected genes. Besides, the primer sequence (5′-3′) for GAPDH (F: ATGAGGTCCACCACCCTGTT, R: CTCAAGGGCATCCTGGGCTA) and KIF23 (F: AACGACGATGAAGTGGCAGAAGG, R: TCAGGTTTGGGTTCAGTCACAATGG) were designed by TSING KE biological technology (Wuhan, China).

#### 4.2.7. Western Blot

The protein extracted from PASMCs were fractionated by SDS-PAGE (10% polyacrylamide gels) and transferred to polyvinylidene fluoride membranes. The membranes were blocked with 5% skim milk at room temperature for 1 h and were then incubated with KIF23 (1:1000), HMGB1 (1:1000), caspase1 (1:500), caspase3 (1:1000), NLRP3 (1:1000), and β-actin (1:10,000) at 4 °C overnight. After three washes with TBST buffer, the membranes were incubated with horseradish peroxidase-conjugated secondary antibodies and enhanced chemiluminescence reagents.

#### 4.2.8. Detecting the Role of KIF23 in PASMCs Pyroptosis and PAH

We first detected the expression level of KIF23 and pyroptosis-related genes (Caspase1, Caspase3, HMGB1, and NLRP3) in PASMCs between control and MCT groups. Then, we knockdown the expression of KIF23 in PASMCs with shRNA and detected the changes in the expression of pyroptosis-related genes. Lastly, we transfected the sh-NC and sh-KIF23 plasmid into rat pulmonary arteries by airway administration and established the PAH animal model with MCT. We detected the hemodynamic index of rats and the expression level of KIF23 and pyroptosis-related genes in PASMCs again.

#### 4.2.9. Cell Counting Kit-8 Determination of PASMCs Proliferation

CCK-8 was used to determine the effect of shKIF23 on the proliferation of PASMCs. When PASMCs in control and shKIF23 groups grew to 70–80% confluency, we starved cells with serum-free DMEM medium for 24 h. After starvation, we replaced it with DMEM (containing 10% FBS) and added 10 μL of CCK-8 to each well to incubate for 3 h. Then, we determined cell proliferation by measuring absorbance at 450 nm.

## 5. Conclusions

In summary, our study found KIF23 was closely associated with PASMCs pyroptosis and IPAH, inhibition of KIF23 could alleviate the progress of IPAH by suppressing pyroptosis and proliferation of PASMCs. KIF23 regulated the expression of Caspase3, NLRP3, and HMGB1, and they were all involved in the PI3K/AKT and MAPK pathways, which were closely related to KIF23. In a word, our study provided a novel idea for targeting PASMCs pyroptosis to treat IPAH.

## Figures and Tables

**Table 1:** Top 10 hub genes of turquoise module screened out by Cytoscape.

Symble	Gene Name	logFC	Adj. *p*. Value	Symble	Gene Name	logFC	Adj. *p*. Value
CCNA2	cyclin A2	0.90	0.044	TOP2A	DNA topoisomerase II alpha	0.98	0.066
BUB1B	BUB1 mitotic checkpoint serine/threonine kinase B	0.97	0.055	RRM2	ribonucleotide reductase regulatory subunit M2	0.76	0.108
DLGAP5	DLG associated protein 5	1.52	0.004	KIF20A	kinesin family member 20A	1.50	0.002
KIF11	kinesin family member 11	1.02	0.008	CCNB2	cyclin B2	1.02	0.054
TPX2	TPX2 microtubule nucleation factor	0.65	0.107	KIF23	kinesin family member 23	0.87	0.010

**Table 2:** The detailed information of IPAH and control samples in our study.

Database	ID	Type	Sex	Age	Database	ID	Type	Sex	Age
GSE144274	PA1217	Control	Male	45	GSE168905	GSM5172154	Control	Female	57
GSE144274	PA1189	Control	Female	17	GSE168905	GSM5172155	Control	Male	1
GSE144274	CHPT1	Control	Male	27	GSE168905	GSM5172156	Control	Male	24
GSE144274	CHLONZA	Control	Male	51	GSE168905	GSM5172157	Control	Male	45
GSE144274	IPAH4	IPAH	Female	56	GSE168905	GSM5172158	Control	Male	46
GSE144274	IPAH1028	IPAH	Female	32	GSE168905	GSM5172164	IPAH	Female	11
GSE144274	IPAH908	IPAH	Male	41	GSE168905	GSM5172165	IPAH	Female	16
GSE144274	IPAHCC014	IPAH	Male	45	GSE168905	GSM5172166	IPAH	Female	39
GSE168905	GSM5172150	Control	Female	33	GSE168905	GSM5172167	IPAH	Female	56
GSE168905	GSM5172151	Control	Female	36	GSE168905	GSM5172168	IPAH	Male	25
GSE168905	GSM5172152	Control	Female	43	GSE168905	GSM5172169	IPAH	Male	40
GSE168905	GSM5172153	Control	Female	46	GSE168905	GSM5172170	IPAH	Male	53

## Data Availability

Based on reasonable use, data and code may be requested from the corresponding author.

## Supplementary Materials

The following supporting information can be downloaded at: https://www.mdpi.com/article/10.3390/ijms23084436/s1.

## Abbreviations

IPAH	Idiopathic pulmonary arterial hypertension
PRGs	pyroptosis-related genes
PASMCs	pulmonary arterial smooth muscle cells
PCD	programmed cell death
MCT	monocrotaline
WGCNA	weighted gene co-expression network
GS	gene significance
MM	module membership
PAP	pulmonary arterial pressure
MAP	mean arterial pressure
CO	cardiac out
PVR	peripheral vascular resistance
SV	stroke volume
EF	ejection fraction
HR	heart rate
PES	end-systolic pressure
ES	enrichment score
PPI	Protein–protein interaction
GSEA	Gene set enrichment analysis
NC	negative control
CCK-8	Cell Counting Kit-8
