# Supplementary material for: Inhibition of KIF23 Alleviates IPAH by Targeting Pyroptosis and Proliferation of PASMCs
Source: Int J Mol Sci. 2022 Apr 18;23(8):4436. doi: 10.3390/ijms23084436 (PMC9032390; doi:10.3390/ijms23084436)
Supplement: Supplementary file 1 [file ijms-23-04436-s001.zip › ijms-1668343-supplementary.pdf]

supplement files

Table S1. Characteristics of datasets in this study

Dataset	Platform	Date	Sample		Subject	Species
			PAH	Normal		
GSE144274	GPL20301	2020	4	4	PASMCs	Homo sapiens
GSE168905	GPL16791	2021	7	9	PASMCs	Homo sapiens

Table S2. The list of 56 pyroptosis-related genes.

Gene	Gene symbol	Gene	Gene symbol
AIM2	absent in melanoma 2	GZMB	granzyme B
BAK1	BCL2 antagonist/killer 1	HDAC6	histone deacetylase 6
BAX	BCL2 associated X, apoptosis regulator	HMGB1	high mobility group box 1
BNIP3	BCL2 interacting protein 3	IL18	interleukin 18
CARD8	caspase recruitment domain family member 8	IL1B	interleukin 1 beta
CASP1	caspase 1	IL6	interleukin 6
CASP10	caspase 10	IRF7	interferon regulatory factor 7
CASP11	caspase 11	NEXN	nexilin F-actin binding protein
CASP3	caspase 3	NLRC4	NLR family CARD domain containing 4
CASP4	caspase 4	NLRP1	NLR family pyrin domain containing 1
CASP5	caspase 5	NLRP12	NLR family pyrin domain containing 12
CASP6	caspase 6	NLRP2	NLR family pyrin domain containing 2
CASP7	caspase 7	NLRP3	NLR family pyrin domain containing 3
CASP8	caspase 8	NLRP6	NLR family pyrin domain containing 6
CASP9	caspase 9	NLRP7	NLR family pyrin domain containing 7
CD147	basigin	NOD1	nucleotide binding oligomerization domain containing 1
CD274	pdl1 Programmed cell death 1 ligand 1	NOD2	nucleotide binding oligomerization domain containing 2
CRTAC1	cartilage acidic protein 1	PDCD6	programmed cell death 6
ELANE	elastase, neutrophil expressed	PJVK	pejvakin
FGF21	fibroblast growth factor 21	PLCG1	phospholipase C gamma 1
FOXO3	forkhead box O3	PRKACA	protein kinase cAMP-activated catalytic subunit alpha
GPX4	glutathione peroxidase 4	PYCARD	PYD and CARD domain containing
GSDMA	gasdermin A	SCAF11	SR-related CTD associated factor 11
GSDMB	gasdermin B	TET2	tet methylcytosine dioxygenase 2
GSDMC	gasdermin C	TIRAP	TIR domain containing adaptor protein
GSDMD	gasdermin D	TLR4	toll like receptor 4
GSDME	gasdermin E	TNF	tumor necrosis factor
GZMA	granzyme A	TXINP	thioredoxin interacting protein

Table S3 The gene number of each module

black	blue	brown	cyan	darkgreen	darkgrey
539	1168	894	336	173	143
darkred	darkturquoise	green	greenyellow	grey	grey60
179	155	706	383	4	314
lightcyan	lightgreen	lightyellow	magenta	midnightblue	pink
332	284	253	858	334	462
red	royalblue	salmon	tan	turquoise	yellow
614	218	338	375	3125	783

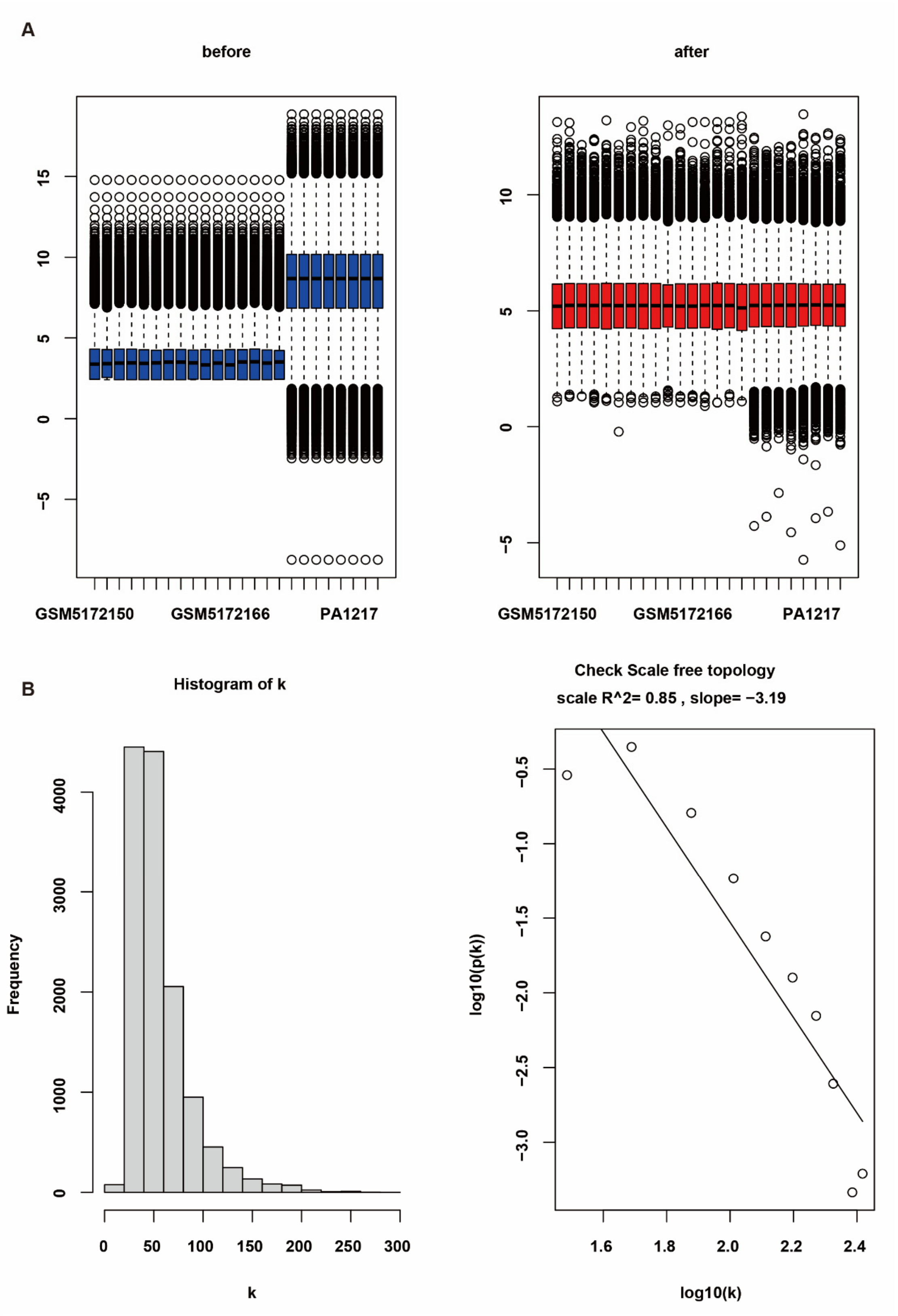

Figure S1 The boxplot of all samples before and after remove batch effect (A) and Scale-free topology when soft-thresholding power β= 5.
